# F180. CANNABINOID 1 RECEPTOR AVAILABILITY & MEMORY FUNCTION IN FIRST EPISODE PSYCHOSIS: A MULTI-MODAL PET-FMRI STUDY

**DOI:** 10.1093/schbul/sby017.711

**Published:** 2018-04-01

**Authors:** Faith Borgan, Mattia Veronese, Owen O’Daly, Tiago Reis Marques, Maria Rogdaki, Oliver Howes

**Affiliations:** 1 King’s College London; 2 Psychiatric Imaging Group, MRC Clinical Sciences Centre, Institute of Clinical Science, Imperial College London

## Abstract

**Background:**

Although memory deficits are a core stable feature of schizophrenia, the neurobiology of these deficits remain poorly understood and unaddressed by current treatments. Converging lines of evidence show that the cannabinoid 1 receptor modulates memory function by altering mitochondrial function as well as synaptic transmission and plasticity. We aimed to investigate the association between memory function and cannabinoid 1 receptor availability, for the first time as far as we’re aware in vivo. We also aimed to investigate the cannabinoid 1 receptor, for the first time as far as we’re aware in first episode psychosis in order to identify if memory function is linked to a cannabinoid 1 receptor dysregulation.

**Methods:**

Sixty-seven volunteers including 32 first episode psychosis patients (28 un-medicated, 4 medicated) and 35 matched healthy volunteers completed the Sternberg working memory paradigm during a functional magnetic resonance imaging (fMRI) scan. A subset of these volunteers including 20 healthy volunteers and 20 first episode psychosis patients (17 un-medicated, 3 medicated) also underwent a dynamic positron emission tomography (PET) scan using a cannabinoid 1 receptor selective radiotracer [11C]MePPEP with arterial blood sampling.

**Results:**

Relative to healthy volunteers, first episode psychosis patients showed a significantly lower availability of cannabinoid 1 receptors in the hippocampus (Hedge’s g=0.6) but showed greater bilateral hippocampal (left: pFWE=0.001; right: pFWE=0.002) and parahippocampal (left: pFWE=0.005; right: pFWE=0.014). functional activation during memory encoding. Healthy volunteers showed an association between CB1R availability in the hippocampus and mean functional activation in the parahippocampal gyrus during memory encoding (R=.567, p=0.027) but this association was not demonstrated by patients (R=.027, p=0.474). Relative to healthy volunteers, first episode patients also showed a significantly lower availability of cannabinoid 1 receptors in the anterior cingulate (Hedge’s g=0.7) which was positively correlated with cognitive performance on the Wechsler Adult Intelligent Scale digit symbol coding test (R=.519, p=0.006) but inversely associated with the severity of delusional symptoms measured using the Positive and Negative Syndrome Scale (R=-.570, p=0.033).

**Discussion:**

We demonstrate for the first time as far as we’re aware that cannabinoid 1 receptor availability is linked to the neural correlates of memory encoding in healthy volunteers. We also demonstrate that first episode psychosis patients altered hippocampal functional activation during a memory task in the context of a hippocampal dysregulation in the cannabinoid 1 receptor. These findings extend an existing body of literature highlighting the role of the hippocampus in the pathophysiology of psychosis. These findings have implications for the understanding and treatment of memory deficits in schizophrenia.

